# Post-Operative and Mid-Term Renal Function Impairment Following Elective Fenestrated Endovascular Aortic Repair for Complex Aortic Aneurysms: Incidence and Risk Factors Analysis

**DOI:** 10.3390/diagnostics13111955

**Published:** 2023-06-03

**Authors:** Elda Chiara Colacchio, Mariagiovanna Berton, Franco Grego, Michele Piazza, Mirko Menegolo, Francesco Squizzato, Michele Antonello

**Affiliations:** Department of Cardiac, Thoracic, Vascular Sciences and Public Health, Vascular and Endovascular Surgery Section, Azienda Ospedale-Università di Padova, Università di Padova, 35128 Padova, Italy

**Keywords:** fenestrated endovascular aortic repair, acute kidney injury, renal function deterioration

## Abstract

*Background.* The aim of this study was to assess the incidence of two post-operative acute kidney injury (AKI) stages according to the Risk, Injury, Failure, Loss of function, End-stage (RIFLE) criteria in patients undergoing fenestrated endovascular aortic repair (FEVAR) for complex aortic aneurysms. Furthermore, we analyzed predictors of post-operative AKI and mid-term renal function deterioration and mortality. *Methods.* We included all patients who underwent elective FEVAR for abdominal and thoracoabdominal aortic aneurysms between January 2014 and September 2021, independently from their preoperative renal function. We registered cases of post-operative acute kidney injury (AKI) both at risk (R-AKI) and injury stage (I-AKI) according to the RIFLE criteria. Estimated glomerular filtration rate (eGFR) was noted preoperatively, at the 48th post-operative hour, at the maximum post-operative peak, at discharge, and then during follow-up approximately every six months. Predictors of AKI were analyzed with univariate and multivariate logistic regression models. Predictors of mid-term chronic kidney disease (CKD) (stage ≥ 3) onset and mortality were analyzed using univariate and multivariate Cox proportional hazard models. *Results.* Forty-five patients were included in the present study. Mean age was 73.9 ± 6.1 years and 91% of patients were males. Thirteen patients (29%) presented with a preoperative CKD (stage ≥ 3). Post-operative I-AKI was detected in five patients (11.1%). The aneurysm diameter, thoracoabdominal aneurysms and chronic obstructive pulmonary disease were identified as predictors of AKI in univariate analysis (OR 1.05, 95% CI [1.005–1.20], *p* = 0.030; OR 6.25, 95% CI [1.03–43.97], *p* = 0.046; OR 7.43, 95% CI [1.20–53.36], *p* = 0.031; respectively), yet none of these factors were significative on multivariate analysis. Predictors of CKD onset (stage ≥3) during follow-up on multivariate analysis were age (HR 1.16, 95% CI [1.02–1.34], *p* = 0.023), post-operative I-AKI (HR 26.82, 95% CI [4.18–218.10], *p* < 0.001) and renal artery occlusion (HR 29.87, 95% CI [2.33–309.05], *p* = 0.013), while aortic-related reinterventions where not significantly associated with this outcome in univariate analysis (HR 0.66, 95% CI [0.07–2.77], *p* = 0.615). Mortality was influenced by preoperative CKD (stage ≥3) (HR 5.68, 95% CI [1.63–21.80], *p* = 0.006) and post-operative AKI (HR 11.60, 95% CI [1.70–97.51], *p* = 0.012). R-AKI did not represent a risk factor for CKD (stage ≥ 3) onset (HR 1.35, 95% CI [0.45–3.84], *p* = 0.569) or for mortality (HR 1.60, 95% CI [0.59–4.19], *p* = 0.339) during follow-up. *Conclusions.* In-hospital post-operative I-AKI represented the main major adverse event in our cohort, influencing CKD (≥ stage 3) onset and mortality during follow-up, which were not influenced by post-operative R-AKI and aortic-related reinterventions.

## 1. Introduction

Complex aortic aneurysms (CAAs) [1] are nowadays increasingly being treated by endovascular means in patients with suitable anatomy, since the reported perioperative mortality related to an open surgical approach goes from 6.8% to 14.7% [1,2].

Branched or fenestrated devices (branched endovascular aortic repair—BEVAR—and fenestrated endovascular aortic repair—FEVAR) are employed to preserve the patency of visceral and renal arteries.

Current guidelines of the European Society for Vascular Surgery [3] recommend FEVAR as the preferred approach in patients with juxtarenal aortic aneurysms (JAAs). The literature shows good FEVAR results both in the immediate post-operative period and long-term follow-up, with a technical success rate of 96–97% [4,5], a freedom from a composite endpoint (including reintervention, branch occlusion, stent migration, aneurysm growth, endoleak and spinal cord injury) of approximately 60% at 5 years [4], and a freedom from major reinterventions of 87% at 5 years [6].

Since performing an FB-EVAR involves the insertion of guides, catheters and stents in the renal arteries, one of the major concerns is the development of post-operative renal function impairment. Post-operative acute kidney injury (AKI) is usually reported according to the Risk, Injury, Failure, Loss of function, End-stage (RIFLE) classification [7], and the incidence ranges from 4.7% [8] to 29% [9]. Multiple reports on the cumulative incidence of post-operative AKI after both BEVAR and FEVAR do exist, yet the two techniques could have different impacts on kidney functions [9,10]. Furthermore, authors often report AKI with different criteria, some considering the serum creatinine but not the estimated glomerular filtration rate (eGFR) or the urine output [11], while others only report an eGFR increase > 25% [10,12]. In fact, we feel that literature data about post-operative renal function are heterogeneous [1].

The aim of this work is to report our results about the rates of two post-operative AKI stages (“risk” and “injury”) according to the RIFLE classification [7] in patients undergoing elective FEVAR for CAAs and thoraco-abdominal aortic aneurysms. Furthermore, we analyzed which of the two stages is connected to chronic kidney disease (stage ≥ 3) [13] onset and mortality during follow-up, and we investigated the factors that are predisposing to post-operative AKI.

## 2. Materials and Methods

### 2.1. Patients and Definitions

We performed a retrospective single-center cohort analysis on all consecutive patients undergoing fenestrated endovascular aortic repair (FEVAR) with a custom-made device for complex abdominal aortic aneurysms (CAAs) or thoraco-abdominal aortic aneurysms (TAAs) from January 2014 to September 2021. CAAs were classified according to Oderich et al. [14], while TAAAs were classified according to Safi et al. [15].

Patients were included in the present study independently from their preoperative renal function status. Exclusion criteria were urgent/emergent interventions, physician-modified endografts, aortic dissections, penetrating aortic ulcers or intramural hematomas and patients undergoing BEVAR. Cases with a customized device including both fenestrations and branches were included since all renal arteries were treated with a fenestration, and branches were dedicated to celiac trunk and superior mesenteric artery.

We collected patients’ demographics, cardiovascular risk factors and comorbidities. The Society for Vascular Surgery (SVS) clinical comorbidity score system [14,16] and the American Society of Anesthesiologists score (ASA) were used to define the cumulative perioperative risk.

Reported intraoperative details included the type of endograft (only fenestrations or fenestrations and branches), the number of fenestrations, interventions performed in multiple steps, and the need for adjunctive intraoperative procedures that aimed to obtain technical success. We could not report the amount of employed contrast media, since it was not always correctly measured and registered.

Major adverse events (MAEs) [14] were considered over the first 30 post-operative days, or over the in-hospital stay, and included all-cause mortality, myocardial infarction, respiratory failure requiring > 24 h mechanical ventilation or reintubation, bowel ischemia requiring surgery, major stroke, grade 3 paraplegia and renal function deterioration with eGFR decrease > 50% or newly onset dialysis; we also noted eGFR declines > 25%.

Patients received dual antiplatelet therapy (aspirin 100 mg daily and clopidogrel 75 mg daily) for the first post-operative month. When the patient was already taking anticoagulation preoperatively, we added a single antiplatelet therapy (aspirin or clopidogrel) for 30 days.

The first post-operative computed tomography angiography (CTA) was performed before discharge or within the first 30 days. Follow-up was then continued at 3 or 6 months, 12 months and yearly thereafter, along with clinical examination. Imaging consisted of alternating CTAs and duplex ultrasound (DUS). 

### 2.2. Renal Function Monitoring

We selected the Modification of Diet in Renal Disease (MDRD) equation [13] to calculate eGFR, because it does not rely on patient’s weight, which we believe was often underestimated because, in our current practice, the value is provided by the patient itself. 

Chronic kidney disease (CKD) ≥ stage 3 according to the National Kidney Foundation criteria [13] was preoperatively registered. We reported AKI onset for both the “risk” (R-AKI) and “injury” stages (I-AKI) (eGFR decreases > 25% and >50%, respectively) based on the RIFLE criteria [7], in order to analyze the association of both stages with overall survival and CKD ≥ stage 3 onset during follow-up. Unlike standard RIFLE criteria, we only considered eGFR and not urine output, since the latter was not always correctly measured during the in-hospital stay.

Both eGFR and serum creatinine were noted preoperatively, at 24–48 h after the intervention, before discharge, and every six months thereafter. During follow-up, we reported cases of CKD onset with a stage ≥ 3 [13], and cases of eGFR deterioration with decreases both superior to 25% and 50%. If a major renal function decline was detected from laboratory testing, patients were also addressed to a nephrologist.

### 2.3. Endpoints

Primary endpoints were the incidence and risk factors of post-operative I-AKI, and the incidence and predisposing factors of renal function deterioration during follow-up, focusing on CKD ≥ stage 3 onset.

Secondary endpoints were primary technical success, incidence of MAEs, overall survival and risk factors of mortality during follow-up.

### 2.4. Statistical Analysis

Analyses were performed with R, version 4.1.0 (R Foundation for Statistical computing, Vienna, Austria) and Prism GraphPad, version 9.4.1 (GraphPad Inc, San Diego, CA, USA).

Continuous variables were described as mean ± standard deviation (SD) or median and interquartile range (IQR).

Risk factors for post-operative I-AKI onset were detected with univariate and multivariate logistic regression, using a penalized likelihood method based on Firth regression to reduce the small-sample bias [17].

Kaplan–Meier survival estimates were used to analyze overall survival and renal function during follow-up. Cox proportional hazards models were used to evaluate factors related to mortality and CKD (stage ≥ 3) onset during follow-up, also applying the penalized likelihood based on Firth regression [17].

Multivariate models were constructed with independent variables whose association with the outcome variable in a univariate model presented a *p*-value ≤ 0.2.

A two-tailed *p*-value < 0.05 was considered statistically significant.

## 3. Results

From January 2014 to September 2021, 46 patients underwent FEVAR in our vascular and endovascular surgery unit. Forty-five of them were treated for a complex abdominal aortic aneurysm (CAAA) [14] or a TAAA and were therefore included in our analysis. Mean follow-up of post-operative renal function was 30 months (range 1–126 months). Risk factors and comorbidities are summarized in Table 1. Mean age was 73.9 ± 6.1, and 91% of patients were males. Thirteen patients (29%) presented with a preoperative CKD ≥ stage 3 [13]. Pararenal AAA and TAAA represented 44% of the cohort, and 4 patients were treated with a mixed design endograft, including both fenestrations and branches, the latter only dedicated to celiac trunk and superior mesenteric artery. Endografts with at least 3 fenestrations were released in 80% of cases.

Primary technical success occurred in 42 cases (93%), and five patients (11%) required intraoperative adjunctive procedures. One case required brachial access to allow the opening of the aortic bifurcated graft gate, one case required the main body relining with an aortic cuff in order to resolve a type 3 endoleak. In one case a target renal artery dissection was corrected with the release of an adjunctive stent, one case required hypogastric artery embolization and distal landing of the aortic bifurcated graft in the external iliac artery because of a calcific common iliac artery rupture during molding, and one case required an unplanned femoral endarterectomy.

MAEs in the first 30 post-operative days were represented by 2 cases (4.4%) of myocardial infarction, 2 cases (4.4%) of respiratory failure, and 3 cases (6.6%) of grade 3 paraplegia. One patient experienced both paraplegia and I-AKI and died of multi-organ failure during the in-hospital stay. Of the three patients with paraplegia, in two of them this was a consequence of spinal catheter displacement and subsequent spinal cord hemorrhage. Eleven patients (24.4%) experienced R-AKI, and five patients (11.1%) experienced I-AKI, however none underwent hemodialysis.

Table 2 represents univariate and multivariate logistic regressions for factors predisposing to post-operative I-AKI. While in univariate analysis this stage of renal function decline was influenced by aneurysms involving renal arteries’ origin, chronic obstructive pulmonary disease (COPD) and increase in aneurysm diameter, and while being of the male sex was a protective factor, none of these factors showed a significant association with I-AKI when a multivariate analysis was performed. Endografts with a number of fenestrations ≥ 3, endografts without branches (fenestrations only), adjunctive intraoperative procedures and interventions performed in multiple steps did not influence I-AKI onset.

**Figure 1:**
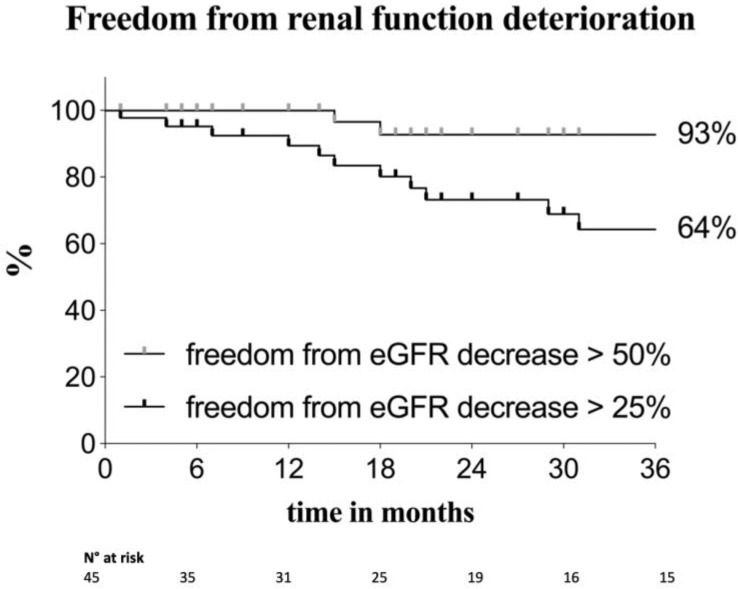
Kaplan–Meier estimates of freedom from renal function deterioration in the overall population. The two curves show patients with a mild renal function decline and patients with an increase of serum creatinine of 2–3 times the baseline or an estimated glomerular filtration rate (eGFR) increase > 50%. Standard error < 10%.

Over the follow-up period, there were four cases of increase of serum creatinine 2–3 times the baseline or an eGFR decrease > 50%, and in 16 patients the serum creatinine increased 1.5–2 times the baseline (or eGFR decreased > 25%). Twenty-five patients had CKD (stage ≥ 3) at the end of the follow-up period: in 13 of these cases, the chronic renal impairment was also present preoperatively. The two survival curves represented in Figure 1 describe the trend in renal function decline during follow-up, for patients experiencing eGFR reduction of both >25% and >50%. At 12, 24 and 36 months, 89.4%, 73.2% and 64% of patients, respectively, were free from any renal function decline or had an eGFR reduction ≤ 25%, while at 36 months, 93% of patients had an eGFR decline ≤ 50% (or no decline).

Fifteen patients (33.3%) had a CKD ≥ stage 3 onset during follow-up. On multivariate Cox proportional hazard models (Table 3), factors associated with CKD (stage ≥ 3) onset were age (HR 1.16, 95% CI [1.02–1.34], *p* = 0.023), post-operative I-AKI (HR 26.82, 95% CI [4.18–218.10], *p* < 0.001) and renal artery occlusion during follow-up (HR 29.87, 95% CI [2.33–309.05], *p* = 0.013), while post-operative R-AKI and reinterventions did not affect this outcome.

**Figure 2:**
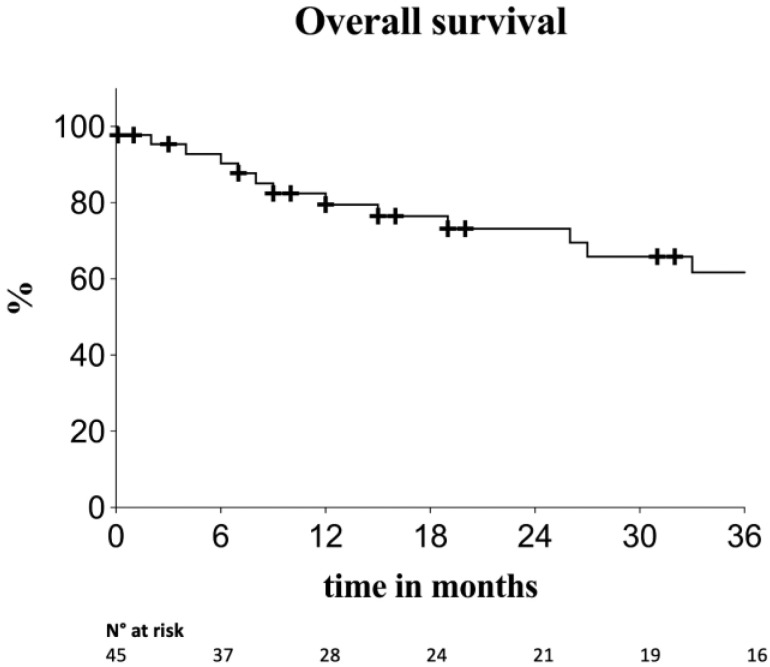
Kaplan–Meier survival estimates for overall survival. Standard error < 10%.

Estimated overall survival was 79.5%, 73.2% and 62% at 12, 24 and 36 months, respectively (Figure 2).

The final multivariate Cox proportional hazard model (Table 4, model adjusted for age and MAEs) showed that CKD ≥ stage 3 (HR 5.68, 95% CI [1.63–21.80], *p* = 0.006) and post-operative I-AKI (HR 11.60, 95% CI [1.70–97.51], *p* = 0.012) were independent predisposing factors for mortality during follow-up.

## 4. Discussion

Acute renal failure represents one of the major complications following endovascular repair for CAAs and TAAAs, ranging from 4.7% [8] to 29% [9]. In current literature, many articles do exist about post-operative renal function analysis, often reporting cumulative results of FEVAR and BEVAR. 

Martin-Gonzalez et al. used the MDRD equation to calculate eGFR [13], and AKI was annotated when an eGFR decrease > 25% occurred (“Risk” stage) [7], with a post-operative rate of 29% and one patient requiring permanent hemodialysis [10]. This was one of the few reports comparing FEVAR and BEVAR, and patients with branched endografts experienced a larger decrease in eGFR values during follow-up [10]. In their next report [9], the authors compared 235 patients treated with BEVAR and 214 patients treated with FEVAR, finding a 12% eGFR decrease rate in the first group and 9% in the second group (not significant when compared), during follow-up. De Lachomette et al. also reported AKI when an eGFR decrease > 25% occurred in patients undergoing FB-EVAR, and found perirenal hematoma and preoperative renal length < 100 mm as factors predisposing to post-operative AKI [12]. Rastogi et al. also analyzed cumulative FB-EVAR results, AKI was defined depending on serum creatinine values, and it was significantly influenced by contrast media amount. Post-operative AKI negatively influenced three years survival both in the standard and the complex EVAR cohort [11].

Of articles only reporting on FEVAR, Saratzis et al. [18] relied on the National Institute of Health and Care Excellence (NICE), and post-operative AKI was defined according to serum creatinine values [19], with a rate of 28%. Furthermore, 4 patients (7%) had an eGFR drop > 30% at 12 months. Sveinsson et al. reported eGFR modifications from the preoperative period to the end of the follow-up (median: 89 months), which was respectively 59.2 ± 14.9 mL/min/1.73 m^2^ and 50.0 ± 18.6 mL/min/ 1.73 m^2^. In the study of Verhoeven et al., patients with a post-operative renal function deterioration (>30% from pre-operative values) represented 5% of the cohort, and one patient required permanent hemodialysis [5]. 

We believe there is a heterogeneity of AKI measuring methods in the current literature. In fact, the latest reporting standards [14] suggest the consideration of renal function deterioration as a MAE when an eGFR decrease > 50% or newly onset dialysis occur, yet the majority of articles only report eGFR drops > 25%, or serum creatinine increases. Furthermore, we feel it is not completely clear which values of acute renal function decline are associated with renal function deterioration and mortality during follow-up. 

In our analysis, we aimed to report our results about both post-operative R-AKI and I-AKI. We subsequently assessed which stage was related to CKD ≥ stage 3 onset and mortality during follow-up. Finally, we performed a multivariate analysis in order to detect factors associated with I-AKI.

A moderate post-operative renal function deterioration (R-AKI) was experienced by 11 patients (24.4%), which was in line with literature [10,18]. On univariate analysis, R-AKI did not show a significant association with CKD (≥stage 3) onset during follow-up, thus it was not included in the final multivariate Cox proportional hazard model. Age, I-AKI and renal artery occlusion were significant predictive factors of renal function deterioration during follow-up, while reinterventions did not affect renal function even on univariate analysis. In fact, even if the rate of R-AKI was not negligible, it did not have a major clinical impact, as both renal function deterioration and survival during follow-up were both only influenced by I-AKI. On the multivariate logistic regression, none of the considered independent variables were shown to be a negative prognostic factor for I-AKI, though it is worth noting that multi-step interventions, the need for adjunctive procedures, and endografts with only fenestrations did not influence acute renal failure onset. Unlike other reports [20], preoperative CKD (≥III stage) did not influence I-AKI onset, however our analysis was performed on a small sample, and it could not consider the amount of contrast media, the pre- and post-operative intravenous hydration and the medications taken by the patients.

Our study has many limitations. First of all, its retrospective nature has prevented us from collecting some information, such as the volume of contrast media employed during the interventions, or from performing correct post-operative urine output monitoring. Furthermore, this is a relatively small sample of patients, with a small number of events. Nevertheless, we believe that some valuable information has been provided regarding the clinical impact of two different AKI stages.

## 5. Conclusions

Acute renal failure remains one of the most common complications after FEVAR for complex abdominal or thoracoabdominal aneurysms.

Although in our cohort renal function deterioration has affected up to 24.4% of patients undergoing aneurysm repair with a fenestrated endograft, only an eGFR decrease > 50% (I-AKI) influenced CKD (≥stage 3) development during follow-up. Adjunctive intra-operative procedures and multi-step interventions did not affect post-operative I-AKI onset. CKD onset (≥stage 3) and mortality during follow-up were both negatively influenced by I-AKI, while they were not affected by reinterventions.

Future studies with a larger number of patients and a longer follow-up are advisable in order to draw definitive conclusions.

## Figures and Tables

**Table 1:** Demographics, aneurysm classification and intraoperative details.

Variable	45 Patients N° (%), Mean ± SD, Median (IQR)
Age	73.9 ± 6.1
Male sex	41 (91)
Cigarette smoking	
current	13 (29)
past	17 (38)
Arterial hypertension	36 (80)
Dyslipidaemia	26 (58)
Diabetes mellitus	8 (18)
Chronic kidney disease (stage ≥ 3)	13 (29)
Coronary artery disease	23 (51)
Percutaneous revascularisation	14 (31)
CABG	8 (18)
COPD	9 (20)
SVS total score	0.9 ± 0.4
ASA ≥ 3	38 (84)
Aneurysm diameter	55.6 ± 11.3
Aneurysm classification	
Juxtarenal	26 (58)
Pararenal	9 (20)
TAAA	10 (22) ^a^
Aetiology: atherosclerotic	45 (100)
Multiple steps procedures	7 (15)
Elective setting	45 (100)
Main endograft type	
All fenestrations	41 (91)
Mixed design ^b^	4 (9)
N° of fenestrations	
1	1 (2)
2	8 (18)
3	16 (36)
4	20 (44)
Brachial access	13 (29)
Days of hospitalisation	7.5 (4–17)

IQR: interquartile range; SD: standard deviation; CABG: coronary artery bypass graft; COPD: chronic obstructive pulmonary disease; SVS: Society for Vascular Surgery; ASA: American Society of Anaesthesiologists; TAAA: thoraco-abdominal aortic aneurysm; ^a^ TAAA: one type 1, four type 2, one type 3 and four type 4; ^b^ mixed design: fenestrations and directional branches.

**Table 2:** Univariate and multivariate logistic regression model (with Firth penalization) for post-operative acute kidney injury (AKI) at “injury” stage (I-AKI).

	Univariate		Multivariate	
	OR (95% CI)	*p* Value	OR (95% CI)	*p* Value
Age (per year)	0.98 (0.85–1.14)	0.801		
Male sex	0.09 (0.01–0.75)	***0.027***	0.09 (0.001–1.16)	0.064
Arterial hypertension	0.45 (0.05–5.22)	0.475		
Diabetes mellitus	1.85 (0.16–13.50)	0.570		
COPD	7.43 (1.20–53.36)	***0.031***	2.32 (0.20–21.92)	0.470
CKD ≥ stage 3	0.76 (0.07–4.70)	0.780		
PAAA or TAAA	2.08 (0.36–13.70)	0.405		
TAAA	6.25 (1.03–43.97)	***0.046***	2.009 (0.16–19.51)	0.555
Aneurysm diameter (per mm)	1.05 (1.005–1.20)	***0.030***	1.05 (0.97–1.43)	0.188
≥3 fenestrations	0.78 (0.12–8.58)	0.814		
Endograft with only fenestrations	1.35 (0.11–189.03)	0.839		
Primary technical success	0.19 (0.02–2.46)	0.181		
Adjunctive procedures	2.70 (0.23–19.96)	0.377		
Days of hospitalisation	1.01 (0.97–1.03)	0.440		
Multiple step intervention	1.76 (0.15–11.93)	0.594		

COPD: chronic obstructive pulmonary disease; CKD: chronic kidney disease; PAAA: pararenal abdominal aortic aneurysm; TAAA: thoraco-abdominal aortic aneurysm. The bold-italic values indicate which variables were inserted in the multivariate model.

**Table 3:** Onset during follow-up.

	Univariate		Multivariate	
	HR (95% CI)	*p* Value	HR (95% CI)	*p* Value
Age (per year)	1.08 (0.98–1.20)	***0.100***	1.16 (1.02–1.34)	***0.023***
Male sex	0.47 (0.13–2.55)	0.343		
PAAA or TAAA	0.35 (0.10–1.0006)	***0.050***	0.32 (0.08–1.09)	0.071
TAAA	0.67 (0.13–2.24)	0.548		
≥3 fenestrations	1.84 (0.58–6.87)	0.306		
Endograft with only fenestrations	1.33 (0.39–6.91)	0.675		
Adjunctive procedures	1.56 (0.30–5.33)	0.545		
R-AKI	1.36 (0.45–3.84)	0.569		
I-AKI	10.25 (2.21–43.73)	***0.004***	26.82 (4.18–218.10)	***<0.001***
Multiple step intervention	2.62 (0.64–8.56)	***0.162***	3.32 (0.72–12.81)	0.113
Renal artery occlusion ^a^	9.95 (0.99–54.02)	***0.050***	29.87 (2.33–309.05)	***0.013***
Reinterventions ^a^	0.66 (0.07–2.77)	0.615		

HR: hazard ratio; CI: confidence interval; PAAA: pararenal abdominal aortic aneurysm; TAAA: thoraco-abdominal aortic aneurysm; MAE: major adverse events;; R-AKI: acute kidney injury with eGFR decline > 25%; I-AKI: acute kidney injury with eGFR decline > 50%; ^a^ during follow-up. In the univariate column, bold italic values refer to variables significantly associated with the outcome and/or inserted in the multivariate model; in the multivariate column, bold italic values indicate significant *p*-values.

**Table 4:** Final multivariate Cox proportional hazard model (with Firth penalization) for factors affecting mortality over the follow-up period. The model is adjusted for patients’ age and the composite outcome major adverse events at thirty days. The bold-italic values indicate significative *p*-values.

	Multivariate	
	HR (95% CI)	*p* Value
Preoperative CKD ≥ stage 3	5.68 (1.63–21.80)	***0.006***
Post-operative AKI (eGFR decline ≥ 50%)	11.60 (1.70–97.51)	***0.012***

## Data Availability

The majority of data are already in the “Results” section. Further data are available on request from the corresponding author.

## References

[1] Antoniou G.A., Juszczak M.T., Antoniou S.A., Katsargyris A., Haulon S. (2021). Editor’s Choice-Fenestrated or Branched Endovascular versus Open Repair for Complex Aortic Aneurysms: Meta-Analysis of Time to Event Propensity Score Matched Data. Eur. J. Soc. Vasc. Surg..

[2] Latz C.A., Boitano L., Schwartz S., Swerdlow N., Dansey K., Varkevisser R.R., Patel V., Schermerhorn M.L. (2021). Editor’s Choice-Mortality is High Following Elective Open Repair of Complex Abdominal Aortic Aneurysms. Eur. J. Soc. Vasc. Surg..

[3] Wanhainen A., Verzini F., Van Herzeele I., Allaire E., Bown M., Cohnert T., Dick F., van Herwaarden J., Karkos C., Koelemay M. (2019). Editor’s Choice-European Society for Vascular Surgery (ESVS) 2019 Clinical Practice Guidelines on the Management of Abdominal Aorto-iliac Artery Aneurysms. Eur. J. Soc. Vasc. Surg..

[4] Mastracci T.M., Eagleton M.J., Kuramochi Y., Bathurst S., Wolski K. (2015). Twelve-year results of fenestrated endografts for juxtarenal and group IV thoracoabdominal aneurysms. J. Vasc. Surg..

[5] Verhoeven E.L.G., Katsargyris A., Oikonomou K., Kouvelos G., Renner H., Ritter W. (2016). Fenestrated Endovascular Aortic Aneurysm Repair as a First Line Treatment Option to Treat Short Necked, Juxtarenal, and Suprarenal Aneurysms. Eur. J. Soc. Vasc. Surg..

[6] Oderich G.S., Tenorio E.R.M., Mendes B.C., Lima G.B.B., Marcondes G.B., Saqib N.M., Hofer J.R., Wong J.M., Macedo T.A. (2021). Midterm Outcomes of a Prospective, Nonrandomized Study to Evaluate Endovascular Repair of Complex Aortic Aneurysms Using Fenestrated-Branched Endografts. Ann. Surg..

[7] Bellomo R., Ronco C., Kellum J.A., Mehta R.L., Palevsky P. (2004). Acute renal failure-definition, outcome measures, animal models, fluid therapy and information technology needs: The Second International Consensus Conference of the Acute Dialysis Quality Initiative (ADQI) Group. Crit. Care.

[8] Motta F., Crowner J.R., Kalbaugh C.A., Marston W.A., Pascarella L., McGinigle K.L., Kibbe M.R., Farber M.A. (2019). Outcomes and complications after fenestrated-branched endovascular aortic repair. J. Vasc. Surg..

[9] Gonzalez T.M., Mastracci T.M., Carrell T., Constantinou J., Dias N., Katsargyris A., Modarai B., Resch T., Verhoeven E., Haulon S. (2016). Mid-term Outcomes of Renal Branches Versus Renal Fenestrations for Thoraco-abdominal Aneurysm Repair. Eur. J. Soc. Vasc. Surg..

[10] Martin-Gonzalez T., Pinçon C., Maurel B., Hertault A., Sobocinski J., Spear R., Le Roux M., Azzaoui R., Mastracci T.M., Haulon S. (2015). Renal Outcomes Following Fenestrated and Branched Endografting. Eur. J. Soc. Vasc. Surg..

[11] Rastogi V., de Bruin J.L., Bouwens E., Hoeks S.E., Raa S.T., van Rijn M.J., Fioole B., Schermerhorn M.L., Verhagen H.J. (2022). Incidence, Prognostic Significance, and Risk Factors of Acute Kidney Injury Following Elective Infrarenal and Complex Endovascular Aneurysm Repair. Eur. J. Soc. Vasc. Surg..

[12] de Lachomette M.F., Della N., Maucort-Boulch D., Duprey A., Rosset E., Feugier P., Lermusiaux P., Albertini J.-N., Millon A. (2017). Renal Function after Fenestrated or Branched Endovascular Aortic Repair: The Early Impairment Predictive Factors. Ann. Vasc. Surg..

[13] National Kidney Foundation (2002). K/DOQI clinical practice guidelines for chronic kidney disease: Evaluation, classification, and stratification. Am. J. Kidney Dis..

[14] Oderich G.S., Forbes T.L., Chaer R., Davies M.G., Lindsay T.F., Mastracci T., Singh M.J., Timaran C., Woo E.Y. (2021). Reporting standards for endovascular aortic repair of aneurysms involving the renal-mesenteric arteries. J. Vasc. Surg..

[15] Safi H.J., Winnerkvist A., Miller C.C., Iliopoulos D.C., Reardon M.J., Espada R., Baldwin J.C. (1998). Effect of extended cross-clamp time during thoracoabdominal aortic aneurysm repair. Ann. Thorac. Surg..

[16] Chaikof E.L., Fillinger M.F., Matsumura J.S., Rutherford R.B., White G.H., Blankensteijn J.D., Bernhard V.M., Harris P.L., Kent K., May J. (2002). Identifying and grading factors that modify the outcome of endovascular aortic aneurysm repair. J. Vasc. Surg..

[17] Firth D. (1993). Bias reduction of maximum likelihood estimates. Biometrika.

[18] Saratzis A.N., Bath M.F., Harrison S.C., Sayers R.D., Bown M.J. (2015). Impact of Fenestrated Endovascular Abdominal Aortic Aneurysm Repair on Renal Function. J. Endovasc. Ther..

[19] Ftouh S., Thomas M. (2013). Acute kidney injury: Summary of NICE guidance. BMJ.

[20] D’Oria M., Wanhainen A., Lindström D., Tegler G., Mani K. (2021). Editor’s Choice-Pre-Operative Moderate to Severe Chronic Kidney Disease is Associated with Worse Short-Term and Mid-Term Outcomes in Patients Undergoing Fenestrated-Branched Endovascular Aortic Repair. Eur. J. Vasc. Endovasc. Surg..

